# Distal Pancreatectomy with Celiac Axis Resection (DP-CAR) for Pancreatic Cancer. *How I do It*

**DOI:** 10.1007/s11605-018-3894-7

**Published:** 2018-08-13

**Authors:** Sjors Klompmaker, Ugo Boggi, Thilo Hackert, Roberto Salvia, Matthew Weiss, Hiroki Yamaue, Herbert J. Zeh, Marc G. Besselink

**Affiliations:** 1 Department of Surgery, Cancer Center Amsterdam, Amsterdam UMC, University of Amsterdam, Meibergdreef 9, 1105 AZ Amsterdam, the Netherlands; 2 Division of General and Transplant Surgery, University of Pisa, Pisa, Italy; 3 Department of General, Visceral and Transplantation Surgery, Heidelberg University, Heidelberg, Germany; 4 Department of Surgery, University of Verona, Verona, Italy; 5 Department of Surgery, Johns Hopkins Hospital, Baltimore, MD USA; 6 Second Department of Surgery, Wakayama Medical University, Wakayama, Japan; 7 Department of Surgery, UT Southwestern Medical Center, Dallas, TX USA

**Keywords:** Pancreas, Cancer, Pancreatic cancer, Pancreas surgery, Appleby, Mortality, Morbidity, Pancreatic fistula, Ischemia, Survival, Technique, High volume, DP-CAR

## Abstract

Approximately 30% of all pancreatic cancer patients have locally advanced (AJCC stage 3) disease. A sub-group of these patients—where the cancer only involves the celiac axis—may benefit from distal pancreatectomy with celiac axis resection (DP-CAR). Previous studies have shown that DP-CAR offers a survival benefit to a selected group of patients with otherwise unresectable pancreatic cancer, when performed by experienced pancreatic cancer treatment teams at high-volume centers. This article proposes a standardized approach to DP-CAR, including routine neoadjuvant (FOLFIRINOX) chemotherapy. This approach to selecting patients and performing DP-CAR has the potential to improve short-term outcomes and overall survival in selected patients, but it should be reserved for high-volume centers.

## Introduction

Pancreatic cancer is projected to become the second most common cause of cancer-related deaths in 2030.^1^ Approximately 30% of all pancreatic cancer patients have locally advanced disease, defined as AJCC stage 3, where the tumor extends beyond the pancreas to include the celiac axis or superior mesenteric artery but without distant metastases.^2^ There is a sub-group of these patients for whom only the celiac axis is involved, while the aorta, superior mesenteric artery, and gastroduodenal artery remain tumor-free. In this patient group, a modified version of the Appleby procedure,^3^ known as distal pancreatectomy with celiac axis resection (DP-CAR),^4^ may lead to a margin-negative resection and a median overall survival (16–32 months) comparable to localized pancreatic cancer.^5^–^10^

After DP-CAR, the liver is perfused by retrograde flow from the superior mesenteric artery via the pancreatic head arcade into the gastroduodenal artery. In recent years, several modifications to DP-CAR have been proposed: preoperative hepatic artery embolization,^10^,^11^ left gastric artery embolization,^10^,^12^ left gastric artery preservation,^13^,^14^ left gastric artery reconstruction via the middle colic artery,^15^ hepatic artery bypass reconstruction using an interposition graft,^16^ and even a robot-assisted approach.^17^ Despite these modifications, high 90-day mortality rates after DP-CAR persist, varying between 3.5 and 17%.^5^–^10^

A recent study from our multicenter group found that treatment at a low-volume DP-CAR center (< 1 annually) was associated with increased 90-day mortality when compared to treatment at higher-volume centers (18 versus 6%).^18^ It may be that the higher level of standardized care in high-volume centers contributes to these improved outcomes. Here, we describe our approach to DP-CAR based on the evidence and empirical lessons learned at seven international high-volume DP-CAR centers. We will discuss neoadjuvant chemotherapy, preoperative artery embolization, left gastric artery preservation, and a stepwise approach to open and robot-assisted DP-CAR.

## Technique

**Fig. 1:**
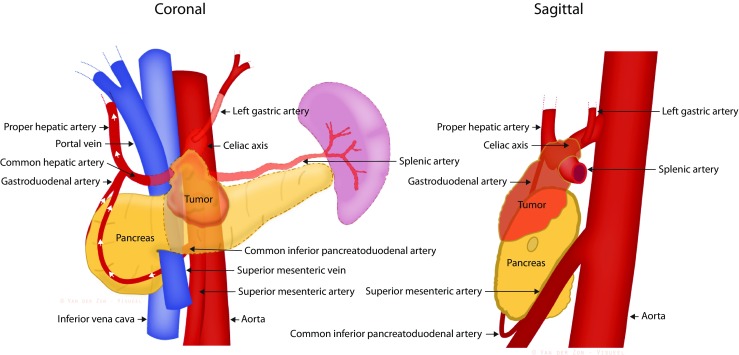
Schematic overview of the anatomy related to DP-CAR. The semi-transparent organs and vessels are resected during DP-CAR. The white arrows represent collateral arterial flow to the liver after resection. This is a schematic representation; the actual origin and insertion of vessels (e.g., left gastric artery) may vary. *Drawing by Van Der Zon Visueel*

Potential candidates for DP-CAR should be discussed in a multidisciplinary setting. DP-CAR should only be considered when distant metastases are ruled out by a recent (i.e., 3–4 weeks) preoperative CT-/MRI-scan. Because both a margin-negative resection and reversed flow to the liver and stomach via the pancreatic head are crucial, the gastroduodenal artery, pancreatic head arcade, superior mesenteric artery, and aorta should all be tumor-free. Concurrent organ- or portal vein involvement (≥ 90–180°) are relative contraindications given the highly invasive nature of the procedure (see Fig. 1). However, if limited vascular involvement is detected intra-operatively, resection and (bypass) reconstruction could be considered.

### Preoperative Chemotherapy

**Fig. 2:**
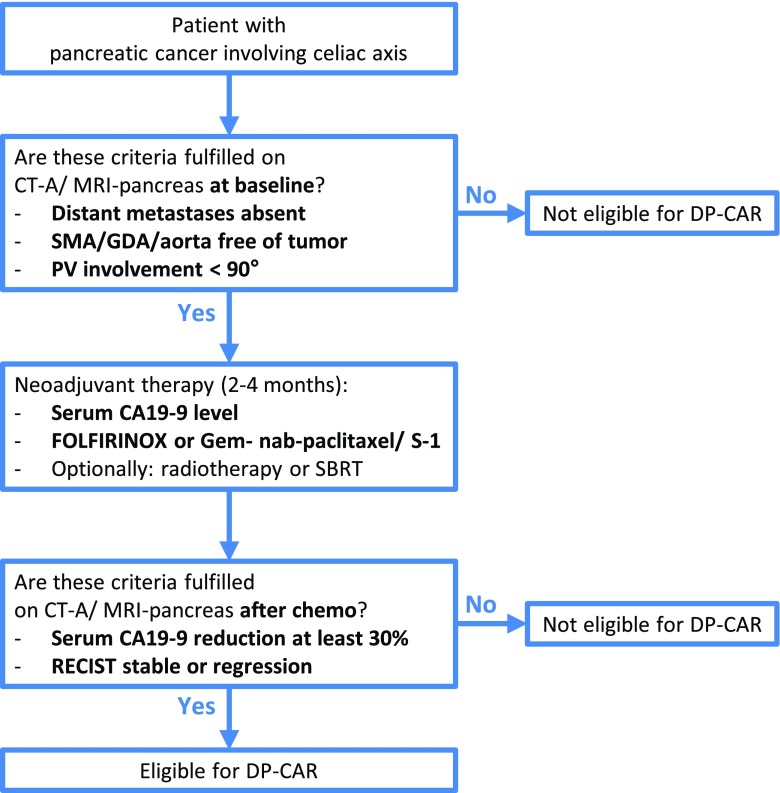
Recommended steps for preoperative work-up. These are consensus recommendations. Individual centers may choose an alternative approach based on their expertise or available treatment modalities. Abbreviations: CT-A, computed tomography angiogram; GDA, gastroduodenal artery; Gem, gemcitabine; MRI, magnetic resonance imaging; SBRT, stereotactic body radiation therapy; PV, portal vein; RECIST, Response Evaluation Criteria in Solid Tumors^26^

Recent studies have shown improved survival in patients with locally advanced pancreatic cancer after neoadjuvant therapy with FOLFIRINOX^19^–^21^ or gemcitabine-based combinations (e.g., S-1 or nab-paclitaxel)^22^–^25^ when compared to upfront surgery or gemcitabine monotherapy. Neoadjuvant therapy is also an established strategy to assess tumor biology. In the scenario where the tumor has progressed prior to preoperative chemotherapy, the risk of mortality associated with DP-CAR probably outweighs the survival benefit. Given the current evidence, we recommend 2 to 4 months of FOLFIRINOX induction chemotherapy prior to re-staging for DP-CAR. Gemcitabine-based combination therapy is a suitable alternative in patients who do not tolerate FOLFIRINOX. If, at re-staging, the tumor is RECIST^26^ stable or even regressive and serum CA 19-9 levels have decreased by at least 30–50%,^27^–^29^ patients are eligible for DP-CAR. See Fig. 2.

### Preoperative Arterial Embolization

**Fig. 3:**
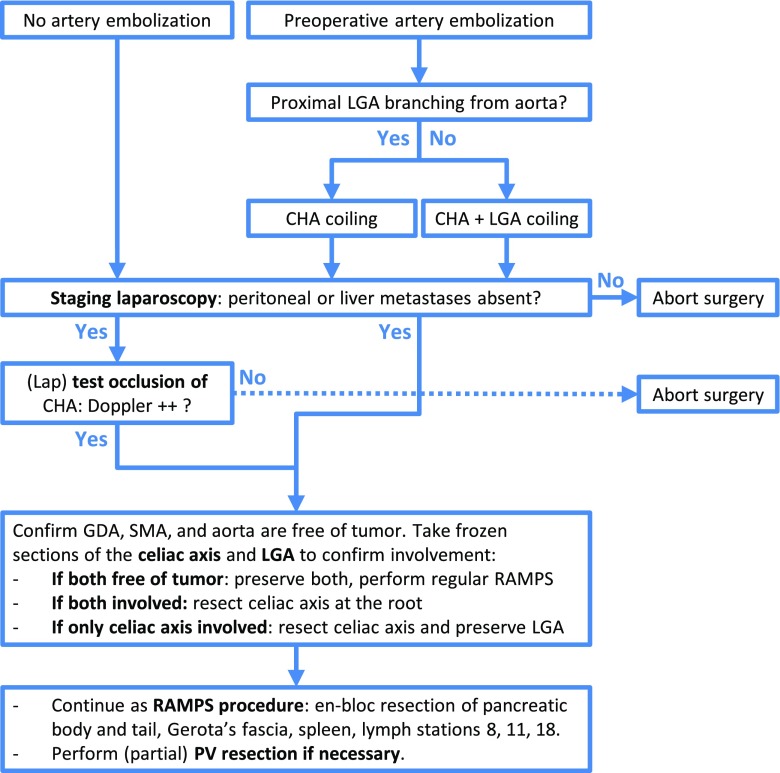
Recommended steps for a standard DP-CAR. These are consensus recommendations for a standardized approach to DP-CAR. Individual surgeons may choose alternative strategies based on their expertise or treatment modalities available at their center. For instance, arterial bypass grafting could be performed (by a vascular surgeon if needed) when no flow is observed upon test occlusion of the common hepatic artery. Abbreviations: CHA, common hepatic artery; GDA, gastroduodenal artery; lap, laparoscopic; LGA, left gastric artery; PV, portal vein; RAMPS, radical antegrade modular pancreato-splenectomy; SMV, superior mesenteric vein

Clear evidence on the effectiveness of preoperative embolization of the common hepatic artery for DP-CAR is lacking.^5^,^6^ Two (WMU, Amsterdam UMC) of the seven centers routinely perform preoperative embolization (i.e., coiling) of the common hepatic artery 2–3 weeks prior to surgery. The coils should be placed by an experienced intervention radiologist who can accurately assess if enough sufficient space remains between the coils and the origin of the gastroduodenal artery.^10^,^11^ Optionally, the left gastric artery is also coiled.^10^,^12^ This treatment is thought to improve collateral flow to the liver and stomach and reduce the rate of postoperative ischemia, at negligible risk of morbidity. In addition, the collateral flow can be assessed preoperatively to prevent futile operations in patients with insufficient flow. If the intention is to preserve the left gastric artery, only the hepatic artery should be coiled. The steps for procedures with and without preoperative artery embolization are presented schematically in Fig. 3.

### Surgical Approach

First, the patient is placed in supine position. Staging laparoscopy is performed during the same procedure, to rule out peritoneal or liver metastases. If no metastases are found, a bilateral subcostal or midline laparotomy is performed, and the liver, peritoneum, and lesser sac are inspected once more for potential metastases. Then, an intra-abdominal ultrasound is optionally performed to confirm celiac axis involvement. It is important to assess the superior mesenteric artery, gastroduodenal artery, aorta, and porto-mesenteric vein by intra-abdominal ultrasound and/or frozen section as CT-/MRI-imaging and visual inspection may be unreliable due to residual inflammation after (FOLFIRINOX) chemotherapy. At this point, an extended DP-CAR could be considered by very experienced surgeons.

Second, Treitz’ ligament is dissected to assess the relation between the tumor and the superior mesenteric artery. An extended Kocher maneuver is performed to assess the inferior vena cava, aorta, and the origin of the celiac axis and superior mesenteric artery, both of which are encircled with vessel loops if technically possible. Again, the relation of the tumor with the superior mesenteric artery and celiac axis is assessed. The pancreas are encircled with vessel loops at the level of the neck, ventral to the portomesenteric vein. Lymph node station 8a (hepatic artery) is dissected as part of the routine lymphadenectomy. Then, the hepatic artery inspected and test-occluded using a bulldog clamp (if not coiled preoperatively), to assess the collateral flow to the liver via the proper hepatic artery with a Doppler probe. If there is adequate collateral flow, the common hepatic artery can later be transected 1 cm proximal to the gastroduodenal artery.

**Fig. 4:**
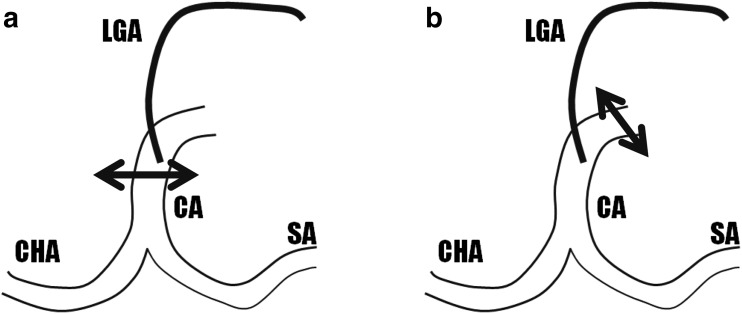
Transection of celiac axis and preservation of the left gastric artery. A schematic overview left gastric artery branching. The double-headed black arrows represent the site of the celiac axis division in case of a: **a** left gastric artery preserving DP-CAR and **b** conventional DP-CAR. Abbreviations: CA, celiac axis; CHA, common hepatic artery; LGA, left gastric artery; SA, splenic artery. Reprinted by permission from Copyright Clearance Center: Springer Nature World J Surg. Preservation of the left gastric artery on the basis of anatomical features in patients undergoing Distal Pancreatectomy with Celiac Axis En-bloc Resection (DP-CAR). Okada et al. Copyright 2014.^14^

Third, the diaphragmatic crus is divided cranially to the celiac axis to clear its origin, the aorta and the left gastric artery. At this point, frozen section may be performed to confirm if the tumor involves the celiac axis and the left gastric artery. If the celiac axis is indeed involved, the hepatic artery is transected as well as the base of the celiac axis using vascular staplers, or suture and clip closure if the adequate distance for stapler positioning cannot be achieved. If the left gastric artery is not involved and its origin is very proximal to the aorta, it may be spared (Fig. 4). The pancreas are divided ventral to the portomesenteric vein using a stapler, surgical blade, or cautery device, similar to a regular distal pancreatectomy for cancer. The left gastric (i.e., coronary) vein and the splenic artery and vein are divided. It is important to preserve the right gastric and gastroepiploic arteries to reduce the risk of gastric ischemia. The dissection continues dorsally onto the superior mesenteric artery which should be freed of all nervous and lymphatic tissue on its left side.

Fourth, the remainder of the procedure follows the steps of a regular RAMPS procedure^30^ using the left renal vein as a landmark. This includes a medial to lateral resection of the anterior renal fascia (Gerota), pancreatic tail, short gastric vessels, and spleen. In an anterior RAMPS, the adrenal gland is left in situ, whereas in a posterior RAMPS, it is included in the resection. Lymphadenectomy is performed according to ISGPS recommendations^31^ and includes stations 11 (supra-pancreatic) and 18 (infra-pancreatic). Lymph station 10 is included in the splenectomy. Although optional, a (partial) portal vein resection (if limited involvement is detected perioperatively) may be carried out as a last step, as long as there is good exposure. If only the left lateral aspect of the portomesenteric confluence is involved, a peritoneal patch could be used.^32^ If not, a segmental resection and graft insertion (autologous vein or synthetic graft) are performed. The pancreatic head remnant typically prevents a tension-free end-to-end anastomosis, which is one of the reasons vascular resections are riskier in DP-CAR compared to standard pancreatoduodenectomy.

Finally, the left pancreas and spleen are removed together with the celiac axis. After confirming adequate flow through the proper hepatic artery, the abdomen is closed and a surgical drain is left in situ at the pancreatic cut margin with extra side holes at the left upper quadrant.

### Robot-Assisted Approach

The robot-assisted approach should only be performed in experienced and very high volume robotic-HPB centers. It also relies on the most up-to-date and high-quality CT- or MRI-imaging. Similar to the open approach, a staging laparoscopy should be performed first. Next, the robot is docked (port set-up outlined elsewhere^7^), the omental bursa opened and the pancreas is tunneled, encircled with vessel loops, and divided using a robotic vascular stapler. The common hepatic artery is transected using a robotic vascular stapler. The dissection continues to identify the superior mesenteric artery, which is traced back to its origin to relocate the celiac axis. The robotic ultrasound should be used frequently to confirm the origins of both the superior mesenteric artery and the celiac axis. At this stage, the splenic artery and left gastric artery and vein are transected distally to their origin at the celiac axis, and medial lymphadenectomy is performed on the superior and right side of the celiac axis. The dissection continues from lateral to medial along the adrenal vein, lifting the spleno-pancreatic complex, including Gerota’s fascia, off of the retroperitoneum. If the portal vein is not involved at this stage, the splenic vein is divided. If it is, the spleno-portal vein resection is performed lastly. Finally, lymphadenectomy of the right side of the celiac axis is performed and the remainder of the celiac axis is transected at its origin using the endovascular linear stapler. The specimens are extracted via a Pfannenstiel incision and a surgical drain is left in situ.

### Postoperative Management

A postoperative enhanced recovery pathway^33^,^34^ is routinely followed. All patients should receive proton pump inhibitors for 6 months. Special attention should be paid to signs of gastric or hepatic ischemia (elevated liver enzymes or serum lactate levels, delayed gastric emptying, or ulcerations) or pancreatic fistula. When ischemia is suspected (abdominal pain, discomfort, abnormal liver enzymes), diagnostic endoscopy or CT-angiography should be considered. Otherwise, postoperative management and follow-up are similar to a regular distal pancreatectomy for cancer, including adjuvant therapy.

## Discussion

This paper proposes a standardized approach to DP-CAR including routine neoadjuvant (FOLFIRINOX) chemotherapy. Previous studies have shown that DP-CAR can be performed safely by experienced teams at high-volume pancreatic surgery centers.^8^,^14^,^18^,^35^ Most importantly, DP-CAR offers a survival benefit to a selected group of patients with otherwise unresectable pancreatic cancer.^36^

As identified in a recent international series of 192 DP-CARs, two major drawbacks of this procedure are the high rates of postoperative major morbidity (27%) and mortality (6–18%). Mortality was most often (52%) associated with gastric- or liver ischemia or to post-pancreatectomy hemorrhaging.^18^ Thus, as DP-CAR is a very invasive procedure, patients who may undergo the procedure should be carefully selected. Outcomes are most favorable if the DP-CAR is limited to resection of the celiac axis, distal pancreas, and spleen. Performing additional organ- or vascular resections, even if technically feasible, is clearly associated with an increased risk of complications and mortality. Furthermore, DP-CAR is a special type of arterial resection for PDAC that needs to be embedded in a multimodality approach, including at least neoadjuvant FOLFIRINOX chemotherapy.^5^

Preventing gastric ischemia is crucial and could be supported by sparing the left gastric artery. The technical feasibility of this approach might be difficult to determine prior to surgery, as CT-imaging is less reliable after FOLFIRINOX chemotherapy.^37^ Future studies should determine whether MRI or PET scans can have an added value in this setting. Intraoperative ultrasound and frozen sections are therefore important to guide decision making. If the celiac axis is free of cancer during surgical exploration, the potential benefit of preoperative hepatic artery embolization obviously disappears. In centers performing arterial reconstructions after resection, hepatic artery coiling may be less attractive as it could hamper an anastomosis between the splenic and hepatic artery.

The optimal surgical approach will of course vary based on local expertise, as well as the quality and timing of recent imaging. Some surgeons with access to very recent high-quality CT may opt for direct transection of the pancreas and vasculature (see *Robot-assisted approach*), whereas others want to assess first if the celiac axis is still involved after FOLFIRINOX treatment and if other major vessels (e.g., superior mesenteric artery, gastroduodenal artery, aorta) are indeed tumor-free. In the future, improved proteomic-/genomic profiling or ex vivo testing of tumor susceptibility to chemotherapy may lead to a paradigm shift in patient selection and preoperative treatment regimens for patients with locally advanced pancreatic cancer.^38^

## Conclusions

A standardized approach to selecting patients and performing DP-CAR following neoadjuvant (FOLFIRINOX) therapy has the potential to improve short-term outcomes and overall survival. However, DP-CAR remains a procedure that should only be done at high-volume pancreatic surgery centers.

## Abbreviations and Acronyms

AJCC: American Joint Committee on Cancer
Amsterdam UMC: Amsterdam University Medical Center
WMU: Wakayama Medical University
DP-CAR: Distal pancreatectomy with celiac axis resection
